# Effect of Berberine on PPAR****α****/NO Activation in High Glucose- and Insulin-Induced Cardiomyocyte Hypertrophy

**DOI:** 10.1155/2013/285489

**Published:** 2013-03-20

**Authors:** Mingfeng Wang, Jia Wang, Rui Tan, Qin Wu, Hongmei Qiu, Junqing Yang, Qingsong Jiang

**Affiliations:** ^1^Department of Pharmacology, Chongqing Key Laboratory of Biochemistry and Molecular Pharmacology, Chongqing Medical University, Chongqing 400016, China; ^2^Department of Pharmacology, Zunyi Medical College, Guizhou 563003, China

## Abstract

*Rhizoma coptidis*, the root of *Coptis chinensis Franch*, has been used in China as a folk medicine in the treatment of diabetes for thousands of years. Berberine, one of the active ingredients of *Rhizoma coptidis*, has been reported to improve symptoms of diabetes and to treat experimental cardiac hypertrophy, respectively. The objective of this study was to evaluate the potential effect of berberine on cardiomyocyte hypertrophy in diabetes and its possible influence on peroxisome proliferator-activated receptor-**α** (PPAR**α**)/nitric oxide (NO) signaling pathway. The cardiomyocyte hypertrophy induced by high glucose (25.5 mmol/L) and insulin (0.1 **μ**mol/L) (HGI) was characterized in rat primary cardiomyocyte by measuring the cell surface area, protein content, and atrial natriuretic factor mRNA expression level. Protein and mRNA expression were measured by western blot and real-time RT-PCR, respectively. The enzymatic activity of NO synthase (NOS) was measured using a spectrophotometric assay, and NO concentration was measured using the Griess assay. HGI significantly induced cardiomyocyte hypertrophy and decreased the expression of PPAR**α** and endothelial NOS at the mRNA and protein levels, which occurred in parallel with declining NOS activity and NO concentration. The effect of HGI was inhibited by berberine (0.1 to 100 **μ**mol/L), fenofibrate (0.3 **μ**mol/L), or *L*-arginine (100 **μ**mol/L). MK886 (0.3 **μ**mol/L), a selective PPAR**α** antagonist, could abolish the effects of berberine and fenofibrate. *N* ^*G*^-nitro-*L*-arginine-methyl ester (100 **μ**mol/L), a NOS inhibitor, could block the effects of *L*-arginine, but only partially blocked the effects of berberine. These results suggest that berberine can blunt HGI-induced cardiomyocyte hypertrophy *in vitro*, through the activation of the PPAR**α**/NO signaling pathway.

## 1. Introduction 

Diabetes mellitus (DM) is a common disease worldwide and its prevalence has increased in recent decades. Patients with type 2 DM are at two-to-five fold higher risk for developing cardiomyopathy, compared with age-matched patients without DM [1, 2]. Diabetic cardiomyopathy is the leading cause of diabetes-related morbidity and mortality [3]; this condition shares many of the characteristics of other types of cardiomyopathy, including ventricular hypertrophy, decreased ventricular diastolic relaxation and reduced peak filling rate; however, it is distinct from other types of cardiomyopathy because it often occurs in the absence of coronary artery disease and hypertension [4, 5]. Ventricular hypertrophy, also known as diabetic cardiac hypertrophy, is the major cardiovascular abnormality in DM patients, and is associated with increased risk for developing congestive heart failure and sudden death [6, 7]. 

**Figure 1:**
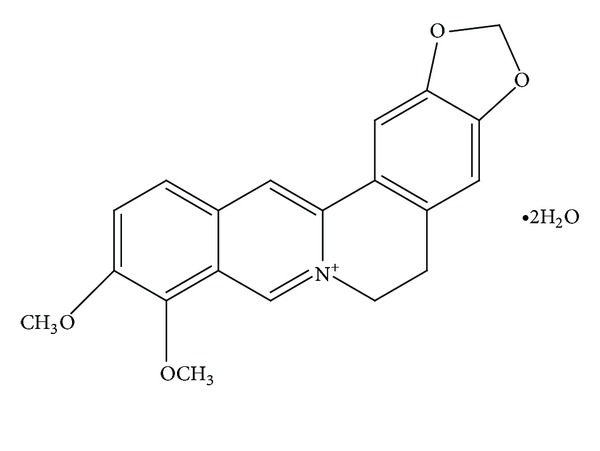
Chemical structure of berberine.

*Rhizoma coptidis* has been used to treat DM in China for centuries. Berberine ([C_20_H_18_NO_4_]^ +^, Figure 1), one of the main ingredients of *Rhizoma coptidis* and *Cortex Phellodendri*, is an isoquinoline alkaloid with multiple pharmacological applications, including its use as an anti-inflammatory, antibacterial, antidiarrheal, and anticancer drug [8–10]. Multiple clinical trials and animal experiments have shown that berberine can improve insulin resistance, correct disorders of lipid metabolism, lower hyperglycemia, and reduce body weight [11]. In the context of the present work, some studies have also demonstrated that berberine has therapeutic potentials in rats with experimental cardiac hypertrophy [12], yet relatively little is known about the potential blunting effect of berberine on diabetic cardiomyopathy, especially on cardiac hypertrophy under diabetic condition.

 Recent studies suggested that the abnormal regulation of peroxisome proliferator-activated receptors (PPARs) was related to the metabolic syndrome, particularly in the advanced stages of DM [13]. PPARs are ligand-dependent transcription factors belonging to the nuclear receptor superfamily. There are three known PPAR isoforms, *α*, *β*/*δ*, and *γ*, which exhibit tissue-specific distribution and ligand-specific effects. In particular, PPAR*α* is abundant in tissues with oxidative energy demands that depend on mitochondrial fatty acid oxidation as a primary energy source, such as the heart [14]. The activation of PPAR*α* may improve diabetic cardiomyocyte hypertrophy, but the downstream molecular mechanisms have not been fully understood. One possible mechanism is through nitric oxide (NO) signaling. *In vitro* and *in vivo *studies have shown that NO could inhibit cardiac hypertrophy induced under various conditions such as hypertension, valvular disease, volume overload, and endothelin-1 challenge [15–17]. Studies that focused on the relaxation effect of PPAR*α* on aortic vessel walls and the cerebral microvasculature have explored the potential therapeutic role of PPAR*α* agonists. This work demonstrated that a PPAR*α* agonist could induce vasodilation, which was mediated through modulating endothelial NO synthase (eNOS) and inducing the release of NO [18, 19]. Our previous experiments have shown that berberine could specifically bind to and activate PPAR*α* [20]; as such the goal of the current study was to determine whether berberine can inhibit high glucose- and insulin-induced cardiomyocyte hypertrophy through the activation of the PPAR*α*/NO signaling pathway.

## 2. Materials and Methods

### 2.1. Chemicals and Reagents

All chemicals and reagents were purchased from Sigma (St. Louis, MO, USA) except berberine (Division of Chinese Material Medical and Natural Products, National Institute for the Control of Pharmaceutical and Biological Products, Ministry of Public Health, Beijing, China) and *L*-arginine and *L*-NAME (Alexis, Lausen, Switzerland).

### 2.2. Primary Neonatal Rat Cardiomyocyte Isolation and Culture

The experimental procedures were approved by the Animal Laboratory Administration Center and Ethics Committee of Chongqing Medical University (SYXK (Chongqing) 2007-0001). Ventricular myocytes from 1- to 3-day-old Sprague-Dawley rats (Animal Laboratory Center of Chongqing Medical University, Chongqing, China) were prepared and cultured for 48 h in Dulbecco's modified Eagle's medium (DMEM) containing 20% fetal bovine serum and 0.1 mmol/L 5′-bromodeoxyuridine [21]. The seeding density was from 0.5 × 10^5^ to 1 × 10^5^ cells/mL for measuring cell surface area, or from 1.5 × 10^6^  to  3 × 10^6^ cells/mL for mRNA extraction, evaluating cellular total protein content, or determining NOS activity and NO concentration in the media. The medium was replaced by serum-free DMEM for a further 48 h before pharmacological treatment. High glucose and insulin (25.5 mmol/L glucose and 0.1 *μ*mol/L insulin, HGI) was used to stimulate the cardiomyocytes. The antihypertrophic effects of berberine (with 99% purity and final concentrations from 0.1 *μ*mol/L to 100 *μ*mol/L), fenofibrate (0.3 *μ*mol/L), and *L*-arginine (100 *μ*mol/L) were studied. MK886 (0.3 *μ*mol/L) or *N* ^*G*^-nitro-*L*-arginine methyl ester (*L*-NAME, 100 *μ*mol/L) were used to investigate the relationship between the antihypertrophic effects of berberine and the PPAR*α*/NO pathway.

### 2.3. Morphometric Analysis

Cellular hypertrophy was evaluated by measuring cardiomyocyte cell surface using a digital image analysis system (Leica QwinV3, Leica Microsystems Ltd., Cambridge, UK). Five random fields (with approximately 10 to 15 cells per field) from every sample were averaged and expressed as *μ*m^2^/cell. All experiments were repeated three times.

### 2.4. Measurement of Cardiomyocyte Protein Content

Collected cardiomyocytes were separated by trypsin and counted; they were then washed three times with ice-cold phosphate-buffered solution (PBS), then homogenized with RIPA lysis buffer (Beyotime, Jiangsu, China) and finally centrifuged at 12 000 g for 20 min at 4°C. The protein concentration in the supernatant was determined with a BCA protein assay kit (Beyotime, Jiangsu, China), and then the protein concentration per 10^6^ cells was calculated.

### 2.5. Real-Time RT-PCR Analysis of mRNA

Total RNA was extracted from cardiomyocytes with Trizol reagent (Takara Biotech Co., Dalian, China), quantified by ultraviolet spectrometric detection (Eppendorf, Germany), and reverse transcribed into cDNA using PrimeScript RT reagent kit (Takara Biotech Co., Dalian, China), according to the manufacturer's instructions. Real-time RT-PCR was performed according to the standard protocol of SYBR *Premix Ex Taq* II (Takara Biotech Co., Dalian, China) on the IQ5 real-time RT-PCR system (Bio-Rad, USA). The standard cycling conditions were 95°C for 8 min, followed by 40 cycles of 95°C for 15 s, annealing for 1 min (atrial natriuretic factor: 61.8°C; PPAR*α*: 60.9°C; eNOS: 59.1°C; *β*-actin: 59.1°C), and 72°C for 40 s. The primers used for SYBR green real-time RT-PCR were synthesized by Takara Biotech Co. (Dalian, China; Table 1). The amount of target gene mRNA relative to the internal control gene, *β*-actin, was calculated using the ΔCt (Ct = cycle threshold) method as follows: the relative expression = 2^−ΔCt^, ΔCt = Ct  (target  gene) − Ct  (*β*-actin). Results of three independent experiments were used for statistical analysis.

### 2.6. Western Blotting Analysis of Protein

The isolated protein (25 *μ*g) from cardiomyocytes was separated by 10% SDS-PAGE and transferred onto polyvinylidene difluoride nylon membranes. The blots were probed with mouse anti-rat PPAR*α* (1 : 700 dilution) or rabbit anti-rat eNOS primary antibodies (1 : 900 dilution; Santa Cruz Biotechnology, Santa Cruz, CA, USA), and then with horseradish peroxidase-conjugated secondary antibodies (1 : 2000 dilution), and visualized using an ECL detection kit (Amersham Biosciences, Piscataway, NJ, USA). The optical densities of the bands were quantified by densitometric analysis performed with a quantitative imaging system (Bio-Rad, USA). All western blot experiments were repeated three times.

### 2.7. NOS Activity Assay

NOS activity in the conditioned medium of cardiomyocytes was measured using the NOS detection kit (Nanjing Jiancheng Bioengineering Institute, Nanjing, China) according to the manufacturer's instructions. The optical density values of the samples were measured at 530 nm with a spectrophotometer. The enzyme activity was expressed as units per mg of protein. Results of six independent experiments were used for statistical analysis.

### 2.8. Nitrite Production Assay

Levels of the NO derivative nitrite were determined in the conditioned medium of cardiomyocytes with the Griess reaction. A nitrite detection kit (Beyotime, Jiangsu, China) was used according to the manufacturer's instructions, and a standard curve using NaNO_2_ was generated for quantification. Briefly, 100 *μ*L of medium or standard NaNO_2_ was mixed with 100 *μ*L of Griess reagent in a 96-well plate. After 15 min, optical density was read in a microplate reader (Tecan Austria Ges.m.b.H) at 540 nm. Results of six independent experiments were used for statistical analysis.

### 2.9. Statistical Analysis

All data in this study were expressed as mean ± SEM. Results were analyzed by one-way ANOVA or *SNK-q* test using the SPSS 13.0 for Windows (SPSS Inc., Chicago, IL, USA). Differences in the mean were considered statistically significant at *P* < 0.05.

## 3. Results

### 3.1. Effect of Berberine on HGI-Induced Cardiomyocyte Hypertrophy

**Figure 2:**
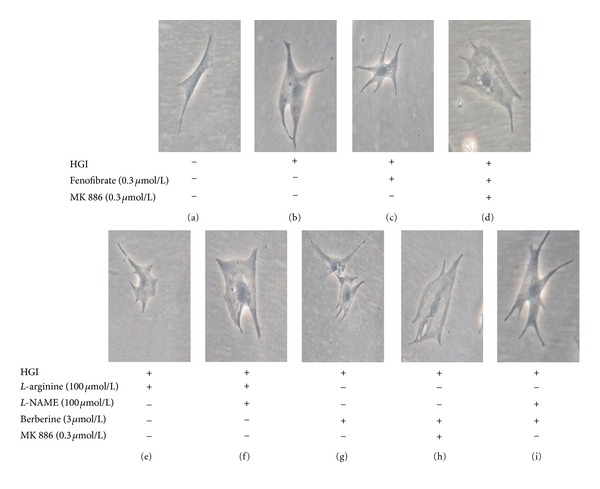
Representative photomicrographs of primary neonatal cardiomyocytes. Stimulation with HGI (25.5 mmol/L glucose and 0.1 *μ*mol/L insulin) for 48 h caused significant hypertrophy (b), compared with the control group (a). Treatment with fenofibrate (0.3 *μ*mol/L) (c), *L*-arginine (100 *μ*mol/L) (e), or berberine (3 *μ*mol/L) (g) inhibited cardiomyocyte hypertrophy induced by HGI. The inhibitory effects of fenofibrate and berberine were completely blocked by cotreatment with MK886, a selective PPAR*α* antagonist (d, h) and the inhibitory effect of *L*-arginine was completely blocked by cotreatment with *L*-NAME, a NOS inhibitor (f). However, the inhibitory effect of berberine was only partially blocked by *L*-NAME (i). “+” or “−”: treatment with or without relevant reagent.

HGI stimulation caused significant cardiomyocyte hypertrophy following a 48 h incubation period, as determined by cell morphometric analysis (Figure 2). The data in Table 2 shows that HGI-stimulation caused a 2.7-fold increase in cell surface and a 2-fold increase in total protein content, compared with those of the corresponding control cells (*P* < 0.05). Treatment with berberine (from 0.1 *μ*mol/L to 100 *μ*mol/L) significantly relieved the changes induced by HGI in a concentration-dependent manner (*P* < 0.05). The IC_50_ (concentration producing a 50% maximal inhibition) for total protein content were 5.5 *μ*mol/L.

 Fenofibrate (0.3 *μ*mol/L), a selective PPAR*α* agonist, had effects similar to berberine (*P* < 0.05). MK886 (0.3 *μ*mol/L), a selective PPAR*α* antagonist, could completely abolish the effects of 3 *μ*mol/L berberine or 0.3 *μ*mol/L fenofibrate (*P* < 0.05). The NO precursor, *L*-arginine (100 *μ*mol/L), also had effects similar to berberine (*P* < 0.05). *L*-NAME (100 *μ*mol/L), a NOS inhibitor, could completely block the effects of *L*-arginine (*P* < 0.05), but only partially block the effect of berberine (3 *μ*mol/L; *P* < 0.05; Figure 2; Table 2). 

HGI-stimulated cardiomyocyte hypertrophy also led to an approximately 4.1-fold induction in the mRNA levels of atrial natriuretic factor (*P* < 0.05), which could be significantly antagonized by berberine, fenofibrate, or *L*-arginine (*P* < 0.05). MK886 abrogated the effects of berberine and fenofibrate on atrial natriuretic factor expression completely (*P* < 0.05), whereas *L*-NAME completely blocked the effect of *L*-arginine, but only partially blocked the effect of berberine (*P* < 0.05; Table 2).

### 3.2. Effects of Berberine on NOS Activity and NO Concentration in the Conditioned Medium of Cardiomyocytes

**Figure 3:**
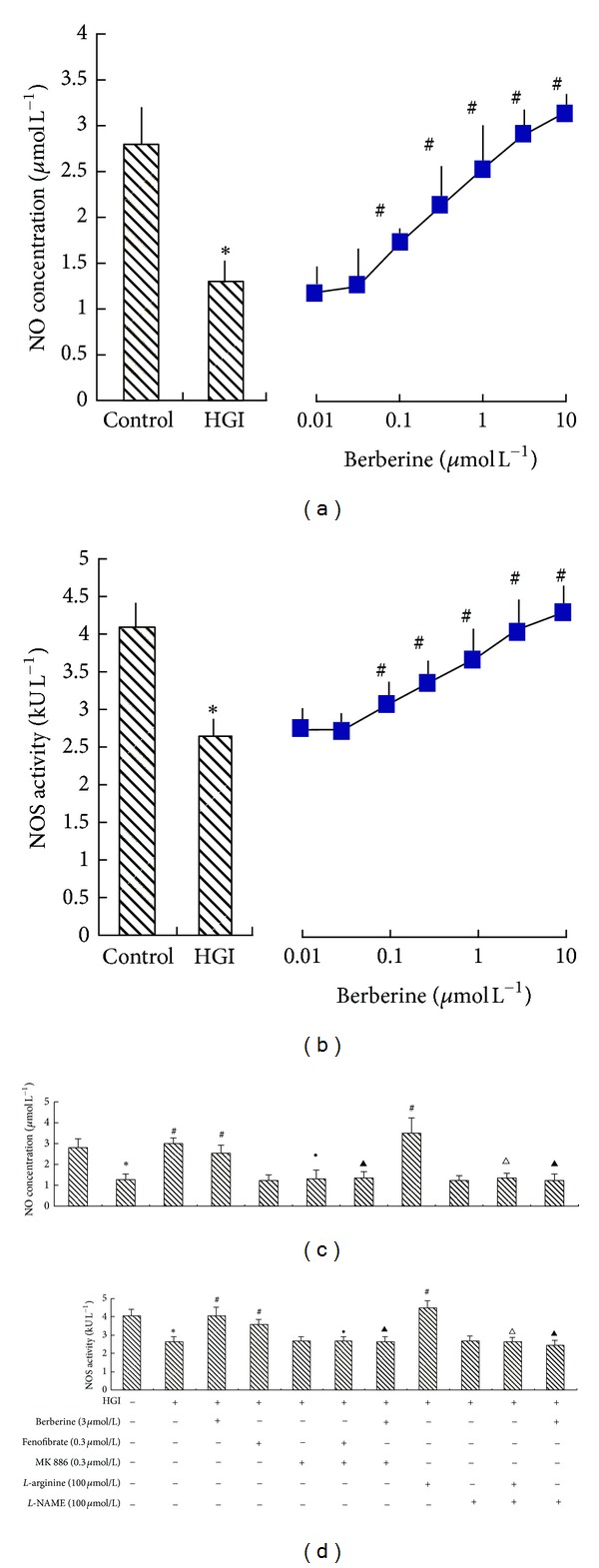
The effect of berberine on NO concentration and NOS activity in hypertrophic cardiomyocytes induced by HGI (25.5 mmol/L glucose and 0.1 *μ*mol/L insulin). Cardiomyocytes were pretreated with various agents for 30 min and then stimulated by HGI for 48 h. The media were then collected for measurement of NO concentration and NOS activity. Levels of NO concentration and NOS activity decreased in the HGI group, and berberine was able to reverse the decrease in NO concentration (a) and NOS activity (b) in a concentration-dependent manner. The effect of various agents, alone or in combination, on NO concentration and NOS activity is shown in (c) and (d). Fenofibrate (0.3 *μ*mol/L), a selective PPAR*α* agonist, or *L*-arginine (100 *μ*mol/L) had effects similar to berberine (3 *μ*mol/L). Moreover, MK886 (0.3 *μ*mol/L), a selective PPAR*α* antagonist, could abolish the effects of both berberine and fenofibrate. *L*-NAME (100 *μ*mol/L), a NOS inhibitor, could abolish the effects of both berberine and *L*-arginine. Results are represented by mean ± SEM of 6 experiments. *P < 0.05 versus control; ^#^ *P* < 0.05 versus HGI; ^●^ *P* < 0.05 versus HGI + fenofibrate (0.3 *μ*mol/L); ^▴^ *P* < 0.05 versus HGI + berberine (3 *μ*mol/L); ^∆^ *P* < 0.05 versus HGI + *L*-arginine (100 *μ*mol/L). “+” or “−”: treatment with or without relevant reagent.

The levels of NOS activity and NO concentration were significantly decreased to 61% and 46% of control levels in HGI-stimulated cardiomyocytes (*P* < 0.05), an effect which was counteracted by berberine in a concentration-dependent manner (0.1 *μ*mol/L to 100 *μ*mol/L; *P* < 0.05). Fenofibrate (0.3 *μ*mol/L) and *L*-arginine (100 *μ*mol/L) had effects similar to berberine (*P* < 0.05), both of which being able to rescue the HGI-induced decrease in NOS activity and NO concentration. MK886 (0.3 *μ*mol/L) was able to abolish the effects of berberine (3 *μ*mol/L) and fenofibrate (*P* < 0.05). Similarly, *L*-NAME (100 *μ*mol/L) could also abolish the effects of berberine (3 *μ*mol/L) and *L*-arginine (*P* < 0.05; Figure 3). 

### 3.3. Effect of Berberine on the Expression of PPAR*α* and eNOS mRNA and Protein in HGI-Stimulated Cardiomyocytes

**Figure 4:**
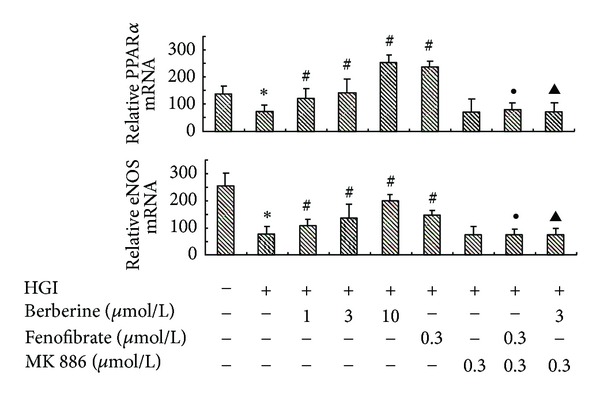
Concentration-dependent effects of berberine on mRNA expression of PPAR*α* and eNOS. Cardiomyocytes were pretreated with various agents for 30 min and then stimulated by HGI (25.5  mmol/L glucose and 0.1 *μ*mol/L insulin) for 48 h. Berberine (1, 3, or 10 *μ*mol/L) markedly restored the decreased PPAR*α* and eNOS mRNA expression level in a concentration-dependent manner. Fenofibrate (0.3 *μ*mol/L) had an effect similar to berberine (*P* < 0.05). MK886 (0.3 *μ*mol/L) abrogated the effects of both berberine and fenofibrate. Results are represented by mean ± SEM of 3 independent experiments. *P < 0.05 versus control; ^#^ *P* < 0.05 versus HGI; ^●^ *P* < 0.05 versus HGI + fenofibrate (0.3 *μ*mol/L); ^▲^ *P* < 0.05 versus HGI + berberine (3 *μ*mol/L). “+” or “−”: treatment with or without relevant reagent.

**Figure 5:**
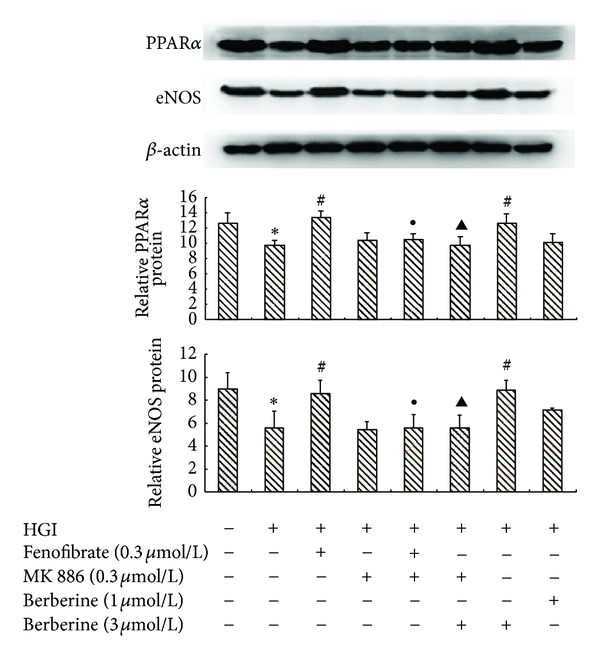
The effect of berberine on the protein expression level of PPAR*α* and eNOS. Cardiomyocytes were pretreated with various agents for 30 min and then stimulated by HGI (25.5 mmol/L glucose and 0.1 *μ*mol/L insulin) for 48 h, and protein expression level was analyzed by Western blot. Berberine (1 and 3 *μ*mol/L) could reverse the HGI-induced decrease in PPAR*α* and eNOS protein expression level. Fenofibrate (0.3 *μ*mol/L) had effects similar to berberine (3 *μ*mol/L). All of the effects of berberine and fenofibrate could be completely abolished by MK886 (0.3 *μ*mol/L). Results are represented by mean ± SEM of 3 independent experiments. *P < 0.05 versus control; ^#^ *P* < 0.05 versus HGI; ^●^ *P* < 0.05 versus HGI + fenofibrate (0.3 *μ*mol/L); ^▲^ *P* < 0.05 versus HGI + berberine (3 *μ*mol/L). “+” or “−”: treatment with or without relevant reagent.

In HGI-conditioned cardiomyocytes, the expression level of PPAR*α* and eNOS decreased by 54% and 26% at the mRNA level, and by 76% and 62% at the protein level, respectively, compared with control (*P* < 0.05). Berberine treatment (1, 3, or 10 *μ*mol/L) markedly elevated the mRNA and protein expression of both PPAR*α* and eNOS in a concentration-dependent manner (*P* < 0.05); the effects of fenofibrate (0.3 *μ*mol/L) similar to berberine were also observed (*P* < 0.05). The rescue effects of berberine and fenofibrate on PPAR*α* and eNOS expression were completely abolished by MK886 (0.3 *μ*mol/L; *P* < 0.05; Figures 4 and 5).

## 4. Discussion

It is well known that DM is characterized by hyperglycemia. This acts as a stimulus for pancreatic beta cells to augment insulin secretion to maintain normal glucose homeostasis; however, long-term hyperglycemia impairs the insulin signaling pathway and depresses the sensitivity to insulin, leading to glucose intolerance and insulin resistance [1]. Therefore, hyperglycemia coupled with hyperinsulinemia develops over time [22], a process which is intimately involved in the pathophysiological process of diabetic cardiomyocyte hypertrophy [23, 24]. In the current study, we used an *in vitro* model to recapitulate diabetic cardiomyocyte hypertrophy in a laboratory setting. The ability of HGI to increase cell surface area, total protein content, and atrial natriuretic factor mRNA expression in rat primary cardiomyocytes suggested that we had induced cardiomyocyte hypertrophy, indicating that high glucose and insulin could mimic the human diabetic condition.

 Berberine has been used as a therapeutic agent in treating many human diseases in Korea, China, and other Asian countries. Although it is one of the most important elements in traditional formulae for the treatment of diabetes in China [25] and has therapeutic effects on chronic heart failure [12], there are few reports on its potential role in the treatment of diabetic cardiomyocyte hypertrophy. In the present investigation, the effect of berberine on HGI-induced cardiomyocyte hypertrophy, for the first time, was evaluated. We observed that berberine effectively inhibited cardiomyocyte hypertrophy caused by HGI in a concentration-dependent manner, suggesting that berberine can effectively inhibit the progression of cardiomyocyte hypertrophy in DM. Notably, our previous experiments showed that berberine could specifically activate PPAR*α* with an EC_50_ of 5.8 *μ*mol/L [20], which was similar to the IC_50_ of berberine (5.5 *μ*mol/L) that inhibited HGI-induced increases in total protein content of cardiomyocytes. We interpreted these similar values to indicate that the antihypertrophic effect of berberine may be related with the activation of the PPAR*α* signaling pathway. 

 PPAR*α* plays an important role in the regulation of lipid synthesis and degradation by virtue of its ability to control key transport proteins and enzymes involved in triglyceride metabolism; therefore, the PPAR*α* signaling pathway may be impaired in diabetes [1]. It is known that transgenic mice with PPAR*α* deletion develop a cardiac hypertrophy mimicking what is observed in the human diabetic condition [26]. Similarly, our results indicated that cardiomyocyte PPAR*α* expression, at both the mRNA and protein levels, was suppressed by HGI stimulation. It is noteworthy that berberine could not only reverse HGI-induced cardiomyocyte hypertrophy, but also markedly upregulate PPAR*α* expression. Meanwhile, MK886 abolished these effects of berberine. In accordance with these findings, treatment of HGI-induced hypertrophic cardiomyocytes with fenofibrate, a PPAR*α* activator, could activate PPAR*α* and improve experimental measures of cardiomyocyte hypertrophy* in vitro*; an effect which was also abolished by MK886. These observations confirm the hypothesis that PPAR*α* is a major intermediate in facilitating the beneficial effects of berberine; however, the downstream molecular mechanisms of PPAR*α* signaling pathway were unclear. 

 NO is synthesized from *L*-arginine by the catalytic reaction of different isoforms of NOS, including neuronal NOS, inducible NOS, and eNOS. Of interest in the current study, is the fact that eNOS is constitutively expressed in cardiomyocytes [27]. In recent years, NO has been emerged as an important regulator of cardiac remodeling and as a potent antihypertrophic mediator [15–17]. Moreover, other studies have revealed that DM impairs eNOS-induced NO production and causes endothelial dysfunction in humans and animals [28–30]. Consistent with these observations, our study also found that eNOS expression in cardiomyocytes, as well as culture medium NOS activity and NO concentration, was significantly decreased by HGI-induced cardiomyocyte hypertrophy, suggesting that this model is related to the reduction of eNOS-modulated NO production. Recently, Yakubu et al. found that the activation of PPAR*α* could increase eNOS expression at the transcriptional and translational levels and further enhance NO production in cerebral microvascular endothelial cells [19]. Similarly, Goya et al. also demonstrated that PPAR*α* activation enhanced NOS expression and activity in isolated endothelial cells [18]. Therefore, we examined the potential crosstalk between PPAR*α* signaling pathway and the eNOS-NO transduction pathway in hypertrophic cardiomyocytes in order to explore the restorative mechanisms of berberine treatment against HGI-induced cardiomyocyte hypertrophy.

 In this regard, our results indicated that both mRNA and protein expression levels of eNOS, as well as culture medium NOS activity and NO concentration, were restored by berberine or fenofibrate treatment in HGI-induced hypertrophic cardiomyocytes. An effect which was correlated with decreased measures of cardiomyocyte hypertrophy and evidence of PPAR*α* activation. Moreover, coadministration of MK886 abolished the stimulatory effects of berberine and fenofibrate. We interpret these results to suggest that NO plays an important role in the antihypertrophic effect of berberine-modulated PPAR*α* activation. Furthermore, the increased NO level was accompanied with enhancement of eNOS mRNA expression, indicating that the activation of PPAR*α* could directly modulate the expression of eNOS. It is intriguing to compare the effects of berberine with NO donors, such as *L*-arginine. Both berberine and *L*-arginine attenuated HGI-induced cardiomyocyte hypertrophy, as well as increased eNOS mRNA expression, NOS activity, and NO concentration. Furthermore, the effects of berberine and *L*-arginine could be abolished by the NOS inhibitor, *L*-NAME; however, it is noteworthy that *L*-NAME did not completely block berberine-mediated attenuation of markers of HGI-induced cardiomyocyte hypertrophy. Therefore, the actions of berberine may not be totally dependent on the NO synthetic pathway; as such the relationship between the effects of berberine and other transduction pathways needs further investigation.

 In conclusion, berberine can inhibit HGI-induced cardiomyocyte hypertrophy, which we consider an* in vitro* model of diabetic cardiomyocyte hypertrophy. Our mechanistic studies reveal that berberine acts via the activation of the PPAR*α* signaling pathway which may, at least in part, promote the expression of eNOS, enhance eNOS activity, and result in a beneficial increase in the production of NO. We believe that our findings should stimulate further interest in berberine as potential therapeutic drug against diabetes-associated heart disease, especially on cardiac hypertrophy under diabetic condition. However, many of the aforementioned effects of berberine require further confirmation in appropriate diabetes models *in vivo*, and validation in patients. 

## Figures and Tables

**Table 1:** Oligonucleotide sequences for real-time RT-PCR.

Gene	Forward (5′-3′ orientation)	Reverse (5′-3′ orientation)
ANF	TGACAGGATTGGAGCCCAGAG	TCGAGCAGATTTGCTGTTATCTTC
eNOS	TGCAACAAACCGAGGCAATC	CACCAGCTGGCTGTTCCAGA
PPAR*α*	CTGACATTTGTGACTGGTCAAGCTC	TTTCCAGGTCATCTGCTTCAAGTG
*β*-actin	GGCCAACCGTGAAAAGATGA	CAGCCTGGATGGCTACGTACA

ANF: atrial natriuretic factor; eNOS: endothelial nitric oxide synthase; PPAR*α*: peroxisome proliferator-activated receptor-*α*.

**Table 2:** Effect of berberine on cardiomyocyte hypertrophy induced by high glucose and insulin (HGI).

Group (*μ*mol/L)	Cell surface area	Protein level	ANF mRNA
	(*μ*m^2^/cell; *n* = 3)	(*μ*g/10^6^ cell; *n* = 6)	(*n* = 3)
Control	459.7 ± 64.1	23.1 ± 5.3	30.6 ± 6.4
HGI	1229.6 ± 99.5*	47.3 ± 3.6*	179.4 ± 25.5*
HGI + berberine (3)	711.2 ± 43.0^#^	37.4 ± 2.0^#^	81.8 ± 7.5^#^
HGI + fenofibrate (0.3)	678.9 ± 21.3^#^	33.8 ± 6.0^#^	37.9 ± 9.5^#^
HGI + MK886 (0.3)	1208.8 ± 27.1	49.9 ± 5.9	184.7 ± 22.3
HGI + fenofibrate (0.3) + MK886 (0.3)	1212.4 ± 82.7^●^	50.2 ± 5.1^●^	161.5 ± 36.5^●^
HGI + berberine (3) + MK886 (0.3)	1257.4 ± 29.6^▲^	47.2 ± 3.3^▲^	154.0 ± 17.6^▲^
HGI + *L*-arginine (100)	768.1 ± 49.0^#^	38.6 ± 4.1^#^	80.7 ± 8.7^#^
HGI + *L*-NAME (100)	1213.1 ± 84.0	48.1 ± 3.5	147.3 ± 20.4
HGI + *L*-arginine (100) + *L*-NAME (100)	1199.9 ± 75.7^∆^	48.8 ± 4.9^∆^	163.3 ± 20.8^∆^
HGI + berberine (3) + *L*-NAME (100)	972.4 ± 41.1^▲^	43.9 ± 2.1^▲^	110.0 ± 9.5^▲^

ANF: atrial natriuretic factor; *L*-NAME: *N* ^*G*^-nitro-*L*-arginine-methyl ester. Results are mean ± SEM of *n* independent experiments. *P < 0.05 versus control; ^#^ *P* < 0.05 versus HGI; ^●^ *P* < 0.05 versus HGI + fenofibrate (0.3 *μ*mol/L); ^▲^ *P* < 0.05 versus HGI + berberine (3 *μ*mol/L); ^∆^ *P* < 0.05 versus HGI + *L*-arginine (100 *μ*mol/L).

## Abbreviations

DM: Diabetes mellitus
PPAR*α*: Peroxisome proliferator-activated receptor-*α*
NO: Nitric oxide
HGI: High glucose and insulin
NOS: Nitric oxide synthase
PPARs: Peroxisome proliferator-activated receptors
eNOS: Endothelial nitric oxide synthase
DMEM: Dulbecco's modified Eagle's medium
*L*-NAME: *N* ^*G*^-nitro-*L*-arginine methyl ester
PBS: Phosphate-buffered solution.
